# Circulating Matrix Metalloproteinases in Infective Endocarditis: A Possible Marker of the Embolic Risk

**DOI:** 10.1371/journal.pone.0018830

**Published:** 2011-04-14

**Authors:** Franck Thuny, Gilbert Habib, Yvan Le Dolley, Matthias Canault, Jean-Paul Casalta, Monique Verdier, Jean-François Avierinos, Didier Raoult, Jean-Louis Mege, Pierre-Emmanuel Morange, Marie-Christine Alessi

**Affiliations:** 1 Département de Cardiologie, Hôpital de la Timone, Assistance Publique-Hôpitaux de Marseille, Université de la Méditerranée, Marseille, France; 2 Unité de Recherche sur les Maladies Infectieuses et Tropicales Emergentes, Centre National de la Recherche Scientifique (CNRS), Unité Mixte de Recherche 6236, Faculté de Médecine de Marseille, Université de la Méditerranée, Marseille, France; 3 Laboratoire d'Hématologie, Hôpital de la Timone, Marseille France, Institut National de la Santé et de la Recheche Médicale (INSERM), Unité Mixte de Recherche 626, Faculté de Médecine de Marseille, Université de la Méditerranée, Marseille, France; Massachusetts General Hospital, United States of America

## Abstract

**Background:**

Embolic events (EE) in infective endocarditis (IE) are caused by fragmentation of vegetations or valvular tissue. Vegetation length is considered to be the most potent predictor of EE, but does not take into account the degree of friability of the vegetation and of the surrounded infected tissue. Matrix metalloproteinases (MMPs) are enzymes involved in degradation of matrix extracellular components and play a role in the pathophysiology of IE. We aimed to determine whether, in addition to the vegetation size, circulating MMPs could provide accurate predictive value of embolism in IE.

**Methods:**

Among 145 patients referred for a native valve IE, we prospectively included 16 patients who experienced EE during antibiotic therapy (new-EE) and 30 patients without new-EE and treated without valvular surgery. A control group of 38 patients with a degenerative valvular heart disease was also included. In addition to clinical, microbiological and echocardiographic assessment, blood MMPs and their inhibitors were assayed in all patients at admission.

**Results:**

MMP-9 serum level was significantly higher in patients with new-EE compared to controls (median [interquartile range]; 250 ng/mL [175–455] *vs.* 111 ng/mL [70–144], respectively; p<0.0001) and patients with no new-EE (250 ng/mL [175–455] *vs.* 138 ng/mL [95–232]; p<0.01). A higher MMP-9 activity in patients who experienced new-EE was further confirmed by gelatin zymography analysis. Circulating MMP-9 remains a predictor of new-EE after adjustment for vegetation length and other potential confounders. This parameter provided incremental predictive value over vegetation measurements.

**Conclusions:**

MMP-9 serum level is associated with the risk of embolism during IE. This marker might help physicians in the management of the disease, but further propspective studies are need to confirm these preliminary results.

## Introduction

The endocardial involvement in infective endocarditis (IE) results in tissue damages and direct contact between blood and the subendothelial host components including proteins of the extra-cellular matrix, thromboplastin, and tissue factor, which trigger blood coagulation and development of vegetations [1], [2]. Valvular endocardium can be severely damaged by pathogens toxins and mediators of the host inflammatory response. Then, tissue remodeling and neo-angiogenesis components (proteolysis and chemotaxis) destroy progressively the valve, increasing the risk of valvular insufficiency, abscesses and cardiac embolization [3].

Emboli are believed to be caused by fragmentation of both vegetations and surrounded valvular tissue. These embolic events (EE) are a frequent and life-threatening complication of IE occurring in 20% to 80% of patients [4], [5], [6], [7], [8]. Currently, the risk of embolism after diagnosis remains about 6% to 21% despite a recommended management including a rapid institution of antimicrobial treatment in all patients and valvular surgery in those with higher risk [5], [6]. Although vegetation length is considered as the most potent predictor of EE, it still remains controversial because it does not take into account the degree of friability of the vegetation and the fragility of the surrounded infected tissues [9]. Thus, the risk of emboli might be determined by the intensity of the coagulation/fibrinolysis activity or by the local inflammatory response and tissue remodeling.

Matrix metalloproteinases (MMPs) constitute a family of endopeptidases having in common the presence of zinc atom in their active site, a Ca^2+^ dependency for their activity, and the ability to interact with specific tissue inhibitors of metalloproteinases (TIMPs) to form enzymatically inactive complexes. MMPs are synthesized as enzymatically inactive precursors by a variety of parenchymal, connective tissue, and inflammatory cells. They show a wide range of specificity for different substrates, including native and partially degraded fibrillar collagens, basement membrane collagens, proteoglycans, elastin, and fibronectin. MMPs, alone or in concert with the plasminogen/plasmin system, are involved in the degradation of extra-cellular matrix components, a requirement for cell migration and tissue remodeling, which play an essential role in many pathological processes such as degenerative valvular heart diseases and endocarditis [10], [11]. Recently, we showed, in a transcriptional profile analysis, a significant up-regulation of many MMPs genes in the cardiac valves during native IE in comparison with degenerative heart valve diseases [3].

Therefore, we aimed to analyze the role of MMPs and TIMPs on the subsequent risk of embolism during IE in addition to the vegetation size.

## Methods

### Ethics Statement

Written informed consent was obtained from all participating patients, as required by the institutional review board under an approved protocol (Comité de Protection des Personnes Sud Méditerranée V, number A00114-51).

### Patients and Controls

From January 2005 to April 2008, all consecutive patients admitted in the Department of Cardiology with the diagnosis of definite native valve IE, according to the modified Duke criteria [12], were eligible for the study entry. Blood cultures and initial clinical, and echocardiographic were systematically performed in all patients at baseline (before the initiation of adequate antibiotic therapy). All patients were followed during their hospital stay and were divided into two groups according to the occurrence of EE during the hospitalization after the beginning of the adequate antibiotic therapy (new-EE). Diagnosis of new-EE was based on clinical and computed tomography scans data. Specific diagnosis of cerebral embolism was confirmed by an experienced neurologist during the clinical course. Since repeated imaging investigations were not systematically performed, silent EE were not included. Cutaneous manifestations or emboli occurring after surgery were not included. The exclusion criteria were: age <18 years, pregnancy, patients under anticoagulation or antiplatelets treatment, patients with abnormalities of hemostasis, and patients who underwent blood samples collection for MMPs and TIMPs assays >48 hours after the beginning of adequate antibiotic therapy. Since valvular surgery suppresses the risk of IE-related embolism, the patients of the no new-EE group who were operated on during the course of antibiotic therapy were excluded from the analysis.

The control group consisted of patients with degenerative valvular heart diseases referred to our Department before cardiac surgery during the same period. We planned to include a similar number of controls than that of IE patients.

### Clinical, Biological and Echocardiographic Data

The following clinical and biological parameters were prospectively collected at baseline: age, sex, diabetes, history of cancer, comorbidity [13], heart failure (HF), previous EE, serum creatinine, C-reactive protein (CRP), hemoglobin concentrations, white cells and platelets counts. Trans-thoracic and trans-esophageal echocardiography (TTE and TEE) were performed at baseline in all cases, as previously reported [4]. Echocardiographic data included the presence and maximal length of vegetation [6]. Measurements of vegetation length were performed in various planes by TTE and TEE, and maximal length was used for analysis. In the presence of multiple vegetations, the largest length was used for analysis. A cut-off value of 10 mm was used because it was demonstrated to be predictive of new-EE in a large population of patients with left and right-sided IE. [6] An abscess was defined as a thickened area or mass with a heterogeneous echogenic or echolucent appearance [14]. Echocardiographic data were stored electronically and used unaltered for subsequent analysis.

### MMPs and TIMPs Assays

Blood samples were collected before or within the 48 hours after the beginning of the adequate antibiotic therapy. MMP-1, MMP-2, MMP-3 and MMP-8 were measured simultaneously as well as TIMP-1, TIMP-2, TIMP-3, TIMP-4 while MMP-9 was measured in a separate test. MMPs and TIMPs were assayed in individual human serum samples using Fluorokine® MultiAnalyte Profiling (F-MAP) kits from R&D Systems (Minneapolis, MN, USA). Kits were run on a Luminex® 100 IS Bioanalyzer (Qiagen, France) according to the kit manufacturer's instructions. Briefly, Fluorokine® MultiAnalyte Profiling kits contained distinct groups of microspheres (each group bearing unique fluorescence intensity and a specific antibody), biotinylated antibodies, and phycoerythrin-conjugated streptavidin. Serum samples were incubated with antibody-coated microspheres binding to specific targets in the serum. Microsphere complexes were washed and incubated with biotinylated antibodies. A final incubation was performed in which phycoerythrin-labeled streptavidin was allowed to bind to biotinylated antibodies present on microspheres. Microspheres were then loaded into a Luminex® 100 Bioanalyzer, which quantifies the amount of phycoerythrin fluorescence present on each of the distinct microsphere groups. At least 50 individual microspheres were counted for each sample, and the median fluorescence intensity was used for subsequent calculations. Each of the microsphere sets was reported by the manufacturer to exhibit less than 0.5% cross-reactivity and interference with the other MMP or TIMP family members studied.

### Zymography

1:20 diluted serum samples (10 µl) were added to non-reducing sample buffer (1/3, v/v) and loaded on to 10% SDS–polyacryamide gels containing copolymerized gelatin (0.1%, w/v) (Sigma Chemicals Co., Saint Louis, MO), and electrophoresis was performed at 75V for 0.5 h then at 100 V for 2 h at 4°C. The gels were then rinsed twice with 2.5% Triton X-100 to remove SDS and renature the MMPs. After a brief wash in distilled water gels were incubated overnight at 37°C in 50 mM Tris–HCl buffer pH 7.6 containing 5 mM CaCl_2_, 200 mM NaCl and Brij 35 (0.02%) with gentle agitation. The gels were stained with Coomassie™ Blue (Sigma) and MMP activities were detected as transparent bands on the blue background. The Scion Image software was used for quantification of the bands after image acquisition (LFG application software) with a CCD camera.

### Statistical Analysis

Categorical variables, expressed as absolute values and percentage, were compared by χ^2^ test or Fisher exact test (two tailed) if the expected count in any cell was <5. Continuous variables, expressed as median (interquartile range), were compared by Mann-Whitney test (comparison between two groups) or Kruskal-Wallis test (comparison of more than two groups) with Dunn's multiple comparisons *post-hoc* test when significant. The association between continuous variables was carried out by using the Spearman rho coefficient.

Odds ratio (OR) and their 95% confidence interval (CI), calculated by logistic regression, were used as measurements of the clinical impact of the predictor variables. For continuous variables, OR were expressed for 1-standard deviation (SD) increase.

Since MMP-9 serum level was significantly associated with new-EE, a receiver operating characteristic (ROC) curve analysis was performed to compare the predictive values of circulating MMP-9 and vegetation length. ROC curve was also used to determine the optimal cut-off value of MMP-9 that best predicted the event. An adjustment for vegetation length (known to be predicted of new-EE)[6] and for the other biological parameters significantly correlated with circulating MMP-9 was performed by logistic regression analysis.

P≤0.05 was considered significant. All analyses were performed with SPSS for Windows 16.0 (SPSS Inc.,Chicago, IL, USA).

## Results

### Patient Characteristics

**Figure 1:**
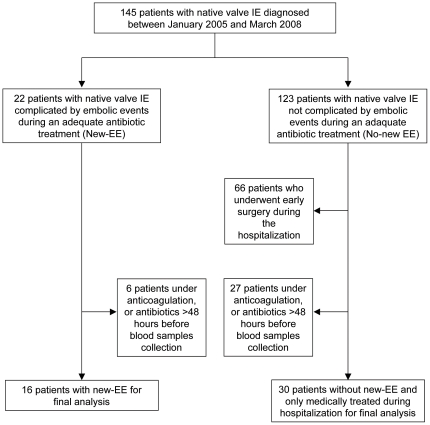
Flow chart showing enrollment of study patients.

During the study period, 145 patients with a definite diagnosis of native valve IE were hospitalized in our department. Forty-six patients were finally enrolled according to the eligibility criteria; 30 patients in the no new-EE group and 16 patients in the new-EE group (Figure 1). New-EE occurred after a median time of 5.5 days (range, 1 to 26) after institution of adequate antibiotic therapy. Sites of embolization were central nervous system (6 cases), pulmonary circulation (4 cases), lower limbs circulation (2 cases), spleen (2 cases), retina (1 case), and coronary circulation (1 case). During the same period, 38 patients with a degenerative valvular heart disease were included in the control group. The characteristics of the three groups are summarized in the Table 1.

**Table 1:** Baseline Characteristics of The Patient Population.

	Controls	No new-EE	New-EE	p^*^	p^†^
	(n = 38)	(n = 30)	(n = 16)		
Age, years	70 (62–79)	71 (61–75)	57 (33–70)	0.005	0.02
Male	16 (42)	24 (80)	10 (63)	0.17	0.20
Cancer	5 (13)	4 (13)	2 (13)	1.0	1.0
Diabetes	3 (8)	8 (27)	1 (6)	1.0	0.13
Comorbidity index >2	20 (53)	20 (67)	6 (38)	0.30	0.06
Heart failure	11 (29)	9 (30)	4 (25)	1.0	1.0
Serum creatinine, µmol/L	92 (72–110)	96(80–138)	87 (73–96)	0.32	0.14
Hemoglobin, g/L	130 (130–140)	113 (99–125)	115 (96–122)	0.002	0.76
White cell count, x10^9^/L	7.4 (6.2–9.0)	8.9 (6.9–11.7)	11.7 (6.1–14.9)	0.04	0.20
Neutrophils, x10^9^/L	4.8 (4.0–6.1)	6.7 (4.9–10.2)	9.5 (4.1–11.7)	0.02	0.24
Platelets, x10^9^/L	238 (205–298)	282 (200–372)	324 (237–381)	0.12	0.53
C-reactive protein, mg/L	4 (2–7)	93 (20–172)	89 (66–169)	<0.0001	0.63
Vegetation length, mm	NA	10 (5–12)	15 (10–20)	NA	0.02
Infected valves					
Aortic	NA	8 (27)	8 (50)	NA	0.11
Mitral	NA	16 (53)	5 (31)	NA	0.15
Right only	NA	7 (23)	4 (25)	NA	1.0
Multivalvular	NA	2 (7)	2 (13)	NA	0.6
Embolic event before diagnosis of IE	NA	12 (40)	3 (19)	NA	0.19
Abscess	NA	3 (10)	2 (13)	NA	1.0
Causative pathogens					
Streptococci	NA	10 (33)	7 (44)	NA	0.48
Staphylococci	NA	9 (30)	4 (25)	NA	1.0
Enterococci	NA	3 (10)	2 (13)	NA	1.0
Others	NA	4 (13)	1 (6)	NA	0.64
No pathogens identified	NA	4 (13)	2 (13)	NA	1.0

Patients with new-EE exhibited larger vegetations compared to patients without new-EE (15 mm [10]–[20] *vs.* 10 mm [5]–[12], respectively; p = 0.02).

### MMPs and TIMPs

**Figure 2:**
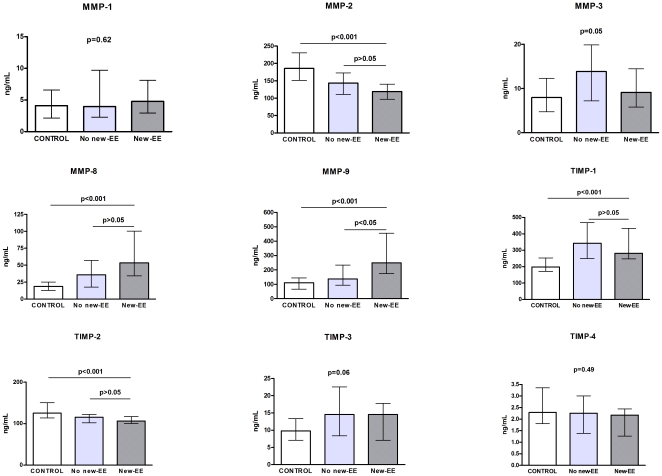
Results of the MMPs and TIMPs serum levels. Only MMP-9 serum level was significantly higher in patients with new-EE compared to controls and patients with no new-EE. Medians and interquartile ranges are shown.

Comparison of MMPs and TIMPs level in serum according to the three study groups are shown in the Figure 2. Only MMP-9 serum level was significantly higher in patients with new-EE compared to controls (250 ng/mL [175–455] *vs.* 111 ng/mL [70–144], respectively; p<0.0001) and patients with no new-EE (250 ng/mL [175-455] *vs.* 138 ng/mL [95–232]; p<0.01). The difference remained significant after exclusion of patients who had experienced an embolic event before the antibiotic treatment and blood collection (p = 0.01).

No significant correlation was observed between MMP-9 serum level and CRP (rho = 0.16; p = 0.28), hemoglobin concentration (rho = 0.02; p = 0.88), creatinine serum level (rho = −0.23; p = 0.12), and platelets count (rho = 0.16; p = 0.29). However, MMP-9 serum level was significantly correlated with neutrophils count (rho = 0.49; p = 0.001), MMP-8 serum level (rho = 0.71; p<0.0001), and vegetation length (rho = 0.38; p = 0.01).

**Figure 3:**
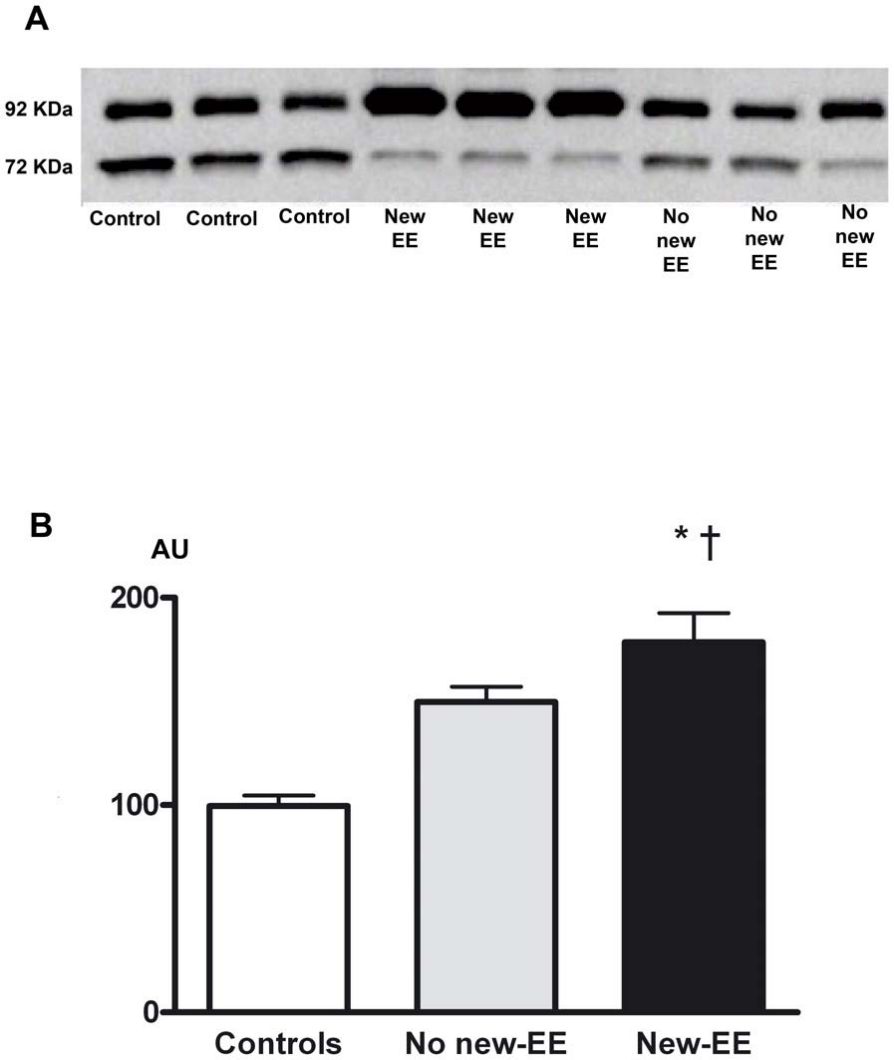
Gelatinolytic activity of MMP-9 (92 kDa) evaluated by in-gel zymography. **A**: Gelatinolytic activity is greater in New-EE patients. **B**: Densitometric analysis of the values of gelatinase activity expressed as arbitrary units (AR). Results are given as means ±SEM. *p<0.0001 *vs.* Controls; †p = 0.05 versus No new-EE patients.

A higher MMP-9 activity in patients who experienced new-EE was further confirmed by gelatin zymography analysis (Figure 3).

### Prediction of New-EE

**Figure 4:**
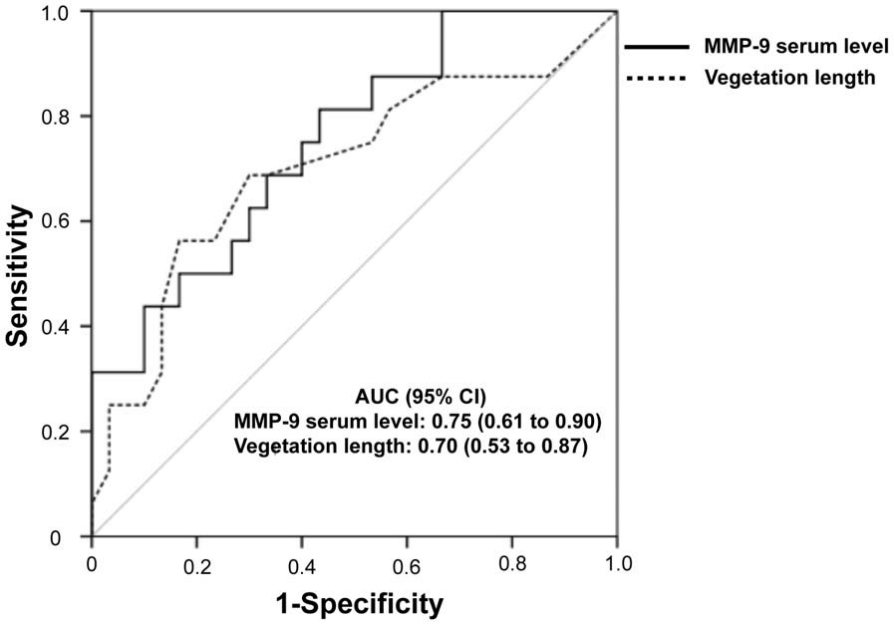
Receiver Operating Characteristic curves analysis using vegetation length and MMP-9 serum level as predictors of new-EE. The area under de curves are given with their 95% confidence interval.

Using single-variable analysis, vegetation length (OR per 1-SD increase  = 2.2; 95% CI, 1.04 to 4.56; p = 0.04) and MMP-9 serum level (OR per 1-SD increase = 3.2; 95% CI, 1.40 to 7.44; p = 0.006) appeared to be predictors of new-EE. Analysis of the ROC curves revealed that the area under the curve was slightly higher for MMP-9 serum level compared to vegetation length (Figure 4). Furthermore, MMP-9 serum level remained predictor of new-EE after adjustment for vegetation length, neutrophils count, and MMP-8 serum level (Table 2). A MMP-9 level threshold of 167 ng/mL was identified as having the highest predictive value for new-EE by the ROC curve analysis. Indeed, new-EE occurred more frequently in patients with MMP-9 serum level >167 ng/mL compared to those with MMP-9 serum level ≤167 ng/mL (50% [13/26] *vs.* 15% [3/20]; p = 0.03).

**Table 2:** Predictors of New-EE by Multivariable Analysis.

	Adjusted OR per 1-SD increase	95% CI	p
MMP-9, ng/mL	3.5	1.19 to 10.34	0.02
MMP-8, ng/mL	0.8	0.32 to 2.13	0.70
Neutrophils, x10^9^/L	0.9	0.40 to 2.08	0.81
Vegetation length, mm	1.9	0.89 to 4.20	0.10

OR = odds ratio; SD = standard deviation; CI = confidence interval.

**Figure 5:**
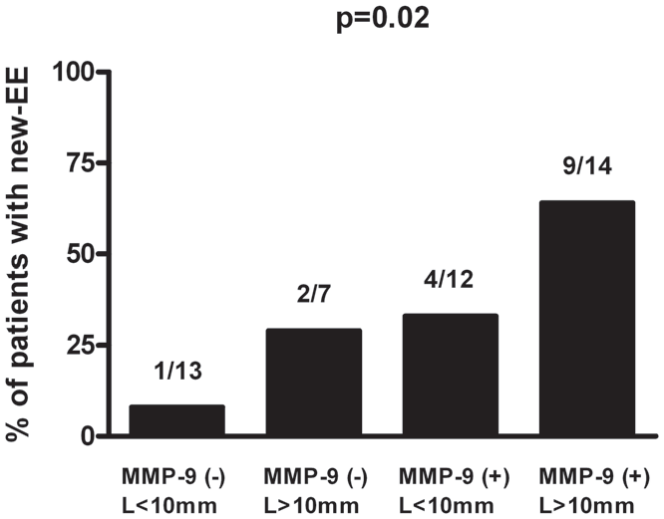
Incidence of new-EE according to the results of vegetation length and MMP-9 serum level. There was an incremental predictive value of MMP-9 over the vegetation length.

MMP-9 assays provided incremental predictive value over vegetation measurements (Table 3). Among the patients with both vegetation >10 mm and MMP-9 serum level >167 ng/mL at admission, the incidence of new-EE under medical treatment was 64% (OR = 6.4; 95% CI, 1.6 to 25.5; p = 0.008) (Figure 5).

**Table 3:** Predictive Values of The Different Risk Stratification Strategies.

	PPV (%)	NPV (%)	OR (95% CI)	p
Vegetation length >10 mm	43	78	4.4 (1.2 to 16.2)	0.03
MMP-9>167 ng/mL	50	85	5.7 (1.3 to 24.1)	0.02
Vegetation length >10 mm	64	78	6.4 (1.6 to 25.5)	0.008
and				
MMP-9>167 ng/mL				

PPV = positive predictive value; NPV = negative predictive value; OR = odds ratio; CI = confidence interval.

## Discussion

The present study is the first demonstrating the possible role of MMP-9 in the pathogenesis of EE during IE. We showed that circulating MMP-9 has an independent predictive value of emboli and provides incremental prognostic information over the echocardiographic data collected at admission.

### Embolic Risk in Infective Endocarditis

During IE the majority of EE involves the central nervous system exposing patients to a higher risk of death [15]. Antibiotics are known to decrease the risk of embolism [5], [6], [16], but in some patients, EE can occur despite this adequate medical therapy. Those high-risk patients have been identified to have more frequently vegetations larger than 10 mm [6]. Therefore, the recent international guidelines have recommended to perform early valvular surgery according to the vegetation length and the presence of previous EE [17], [18], [19]. However, despite this therapeutic strategy based on the vegetation length, the incidence of EE remains between 6% to 21% in the most recent series [5], [6], [16], [20]. This could be explained by a surgical treatment performed too late in patients with large vegetations, and also by the limitations of the vegetation length predictive value. Thus, we decided to initiate the present study in order to identify biomarkers that could help in the tricky decision of surgical indication in prevention of EE. Few previous works attempted to find such a biological parameters. Kupferwasser et al. found that, identification of infection-associated antiphospholipid antibodies in patients with IE was associated with a higher rate of EE. However, one of the limitations in their study was the inclusion in the analysis of EE (9/26) that had occurred before the admission and blood collection [21]. Two other investigations focussed their analysis on the impact of endothelial cell and platelet activation, systemic coagulation activity, and fibrinolytic capacity on the subsequent risk of thromboembolic events. These events were associated with an increased serum level of many markers of thrombus formation but no cut-off value was identified for each of those and their predictive values were not clearly compared with that of vegetation length, which remains the most validated predictor of EE [22], [23].

### MMPs in Infective Endocarditis

Based on the pathophysiological concepts of IE, we aimed to analyze the impact of MMPs on the subsequent risk of embolism because these molecules seem to be involved in many steps of the IE pathogenesis [3], [24], [25], [26]. Moreover, we decided to analyze the relationship between these molecules and the vegetations length.

Matrix metalloproteinases are involved in many normal and pathological processes such as myxomatous valvular heart disease [27], atherosclerosis plaque instability [28], aneurysm development [29], cancer [30], heart failure[31] and infectious diseases [10]. In normal conditions, MMPs are produced by inflammatory (neutrophils and macrophages) and other resident cells. Cytokines and growth factors including IL-1, IL-10, platelet-derived growth factor, and TNF-α have been shown to induce or stimulate MMPs synthesis [31], [32], [33]. The MMPs play a role in infectious diseases when the host immune system is challenged by an invading organism, facilitating the recruitment of leucocytes from the bloodstream for eradication of the pathogen and modulating the inflammatory response [10]. In addition, they participate to the coagulation/fibrinolytic system and thus may play a role in the coagulation/fibrinolytic response to infection [34]. However, the excess of MMP activity following infection may cause immunopathological processes that lead to host morbidity or mortality and favor pathogen dissemination or persistence [10]. Indeed, some studies found an over expression of MMPs in valvular tissues of IE and strongly suggested that MMPs participated to the tissue destruction, layers disruption and neovascularization observed during IE [3], [24], [25]. Moreover, the role of MMPs in embolic events has been demonstrated in many pathological processes such as atherosclerotic plaque rupture[28] and cardiac myxoma [35]. Recently, we showed, in a transcriptional profile analysis, a significant up-regulation of many MMPs genes in the cardiac valves during native IE in comparison with degenerative heart valve diseases [3]. In this latter study, MMP-12 was especially associated with IE, but also MMP-9. In the present work, MMP-12 was not assessed because its serum level is diffcult to obtain in comparison to the others. By demonstrating that circulating MMP-9, also known as 92-kDa gelatinase, was strongly associated with IE-related emboli, our study suggests that an intense local valvular inflammation can lead to MMP-9 overexpression, and then to promote EE by increasing the fragility of the cardiac infected tissue and vegetations. Hence, there may be an increased release of this enzyme into the circulation from the infected tissues themselves or by circulating macrophages or neutrophils involved in the inflammatory response. On the other hand, this increase might represent a marker of end organ ischemia after EE. However, in our study the MMP-9 serum level remained predictive of new-EE after exclusion of patients who had experienced EE before the blood sample collection.

Although we did not find a significant correlation between circulating MMP-9 and CRP, the high level of circulating MMP-9 associated with EE may reflect a more important local inflammatory reaction leading to a high fragility of vegetations and surrounded tisues.

In the present study, we showed a higher prognostic value of MMP-9 and MMP-8 compred to the other metalloproteinases. One explanation would be that, in the context of infectious disease, neutrophils MMP-9 and -8 can be released immediately after stimulation, whereas the others required a longer process of increase gene transcription to drive secretion. Moreover, it has been shown that some pathogens can induce secretion of MMP-9 by host cells, but also activated (cleaved) it by secreting proteolytic enzymes [10]. Finally, the absence of significant changes in TIMP serum level in IE emphasized the shift in the balance of circulating enzymes towards proteolysis and then tissue destruction and embolization.

### Vegetation Length and MMPs in Prediction of Embolism

In accordance with other previous studies [5], [6], we found vegetation length as a predictor of new-EE. Among patients with vegetation length >10 mm at admission, 43% experienced emboli during antibiotic therapy. Besides, MMP-9 serum level was also predictive of new-EE independently from vegetation length and with a higher predictive value. Our results indicate that 50% of the patients with circulating MMP-9 >167 ng/mL at admission experienced new-EE during antibiotic therapy. In addition, we tested, for the first time, the use of an echocardiographic and a biological parameter in the prediction of EE under medical treatment. Thus, the better predictive value was obtained with the combination of the two parameters since 64% of patients with both vegetation length >10 mm and MMP-9 >167 ng/mL experienced EE under medical treatment whereas only 8% had EE in absence of the two criteria. These results suggest that not only the size of vegetation influences the embolic risk but also its instability as well as the friability of the surrounded infected cardiac tissues as determined by MMP-9 activity.

### Limitations

The rates of emboli under IE treatment in the present study were much higher than those previously published because of a specific design. Indeed, in the European multicenter study, the rate of EE under antibiotic therapy was 7.3%, but in this analysis the patients who underwent surgery before the occurrence of EE were included.s[6] Since those patients were excluded in the present work, the rate of new-EE observed is rather representative of the risk of embolism under medical therapy alone than the risk observed if the current recommendations of surgical management are applied. However, using the current study design, we believe that the bias related to surgery performed before the occurrence of a potential EE was limited.

No analysis of MMPs during follow-up was performed, thus, we were unable to establish the time course of MMP-9 activity in the patients with new-EE and the others. In this study, silent EE were not systematically investigated, thus their occurrence could influence our results.

### Clinical Implications and Conclusions

Although recent international guidelines recommend to perform early valvular surgery in patients with large vegetations associated with recurrent emboli or other indications of surgery, the decision to operate can be more difficult in patients with isolated large vegetations. Our study identified circulating MMP-9 as a possible biomarker of the embolic risk. This parameter might be used in association with the vegetation length to predict EE under medical therapy and then to indicate surgery. However, further investigations are required to confirm these preliminary results and to assess their utility in the clinical practice.
